# Delayed spinal extradural hematoma following thoracic spine surgery and resulting in paraplegia: a case report

**DOI:** 10.1186/1752-1947-2-141

**Published:** 2008-05-02

**Authors:** Chandra JKB Parthiban, Shiju A Majeed

**Affiliations:** 1 Kovai Medical Center and Hospital, Coimbatore, Tamilnadu, India

## Abstract

**Introduction:**

Postoperative spinal extradural hematomas are rare. Most of the cases that have been reported occured within 3 days of surgery. Their occurrence in a delayed form, that is, more than 72 hours after surgery, is very rare. This case is being reported to enhance awareness of delayed postoperative spinal extradural hematomas.

**Case presentation:**

We report a case of acute onset dorsal spinal extradural hematoma from a paraspinal muscular arterial bleed, producing paraplegia 72 hours following surgery for excision of a spinal cord tumor at T8 level. The triggering mechanism was an episode of violent twisting movement by the patient. Fresh blood in the postoperative drain tube provided suspicion of this complication. Emergency evacuation of the clot helped in regaining normal motor and sensory function. The need to avoid straining of the paraspinal muscles in the postoperative period is emphasized.

**Conclusion:**

Most cases of postoperative spinal extradural hematomas occur as a result of venous bleeding. However, an arterial source of bleeding from paraspinal muscular branches causing extradural hematoma and subsequent neurological deficit is underreported. Undue straining of paraspinal muscles in the postoperative period after major spinal surgery should be avoided for at least a few days.

## Introduction

Symptomatic spinal epidural (extradural) hematoma (SEH) is rare [1]. A description by Jackson [2] in 1869 is credited as the first official record of an SEH. Since that time, several hundred cases with various origins have been reported in the literature. Most are the result of trauma, anticoagulation therapy, vascular anomalies and blood dyscrasias or occur following spinal epidural procedures and, rarely, spinal surgery. Increased incidence of postoperative SEH (PSEH) is found to occur in people aged over 60, patients on pre-operative non-steroidal anti-inflammatory drugs and those with Rh-positive blood type. Significant intra-operative variables include more than five spinal levels subjected to surgery, a hemoglobin level of less than 10 g/dl and blood loss of more than 1 liter [3]. Repeat surgeries have a higher incidence of PSEH [4]. A venous source of epidural hematoma is well established; however, PSEH from an arterial bleed, also occurring in a delayed fashion, is underreported. We report a case of progressive neurological deficit following a compressive extradural hematoma in a patient who had undergone spinal surgery in the thoracic spine for the excision of a tumor. The source of the bleed was found to be a paraspinal muscular arterial bleed.

## Case presentation

A 34-year-old man presented with a 3-month history of paresthesia of his left foot. On examination, he had impairment of dorsal column sensation in both feet, with intactness of other modalities of sensation, and preserved motor and bladder functions. He had no other comorbidities in the form of diabetes or coagulation disorders. He had not undergone any previous surgical procedures. He was on a short course of gabapentin prior to the diagnosis. A magnetic resonance imaging (MRI) scan revealed an intradural extramedullary lesion arising from the T8 root on the left side compressing the spinal cord.

He underwent surgery for excision of the tumor. Under general anesthesia, a subspinous laminectomy was performed from T7 to T9, and the tumor was approached intradurally on the left side.

In subspinous laminectomy, the interspinous ligament on the distal limit of the laminectomy is cut and the spinous processes of the desired vertebrae are cut using angled bone-cutting forceps and turned proximally by preserving the interspinous ligament on the proximal limit of the laminectomy. On completion of the procedure, the spinous processes are sutured back.

A radicular vessel was seen dorsal to the tumor which was preserved. Intracapsular debulking was performed and the tumor was excised following resection of the dorsal nerve root from which the capsule was seen to arise. The tumor was yellowish-grey and bilobed and was sent for histopathologic examination. The dura was closed in the standard fashion and gel foam and a dural patch were placed epidurally. A subfascial gravity drain was placed. No suction was applied, as is the standard procedure following durotomy. Adequate hemostasis was achieved. The patient recovered from anesthesia with normal neurology. He was progressing well for the first 2 days following surgery. He was kept on log rolling (i.e. simultaneous turning of shoulder, back and pelvis with assistance) and assisted with movements of the limbs.

On the first postoperative day (POD), the drain collected 150 ml which was predominantly altered blood. During the second POD, the drain collected 200 ml which contained cerebrospinal fluid (CSF) mixed with altered blood. On the third POD, drain was 150 ml of clear CSF, indicating that hemostasis was adequate in the immediate postoperative period.

After 72 hours, our patient experienced stabbing pain in his back with radiation to the legs following repeated unassisted voluntary twisting of the body, despite being advised of the need for strict log rolling. A fresh streak of blood was observed in the drain by the attending clinician when the pain was experienced. Gradually, the patient complained of progressive numbness of both his lower limbs with deterioration in motor power to grade 3 with sensory impairment from T10 downwards. The time interval from the onset of pain to neurological deterioration was 2 hours. His motor power deteriorated further to grade 0, with complete anesthesia below T10, within a further short period of about 1 hour. By this time, the drain showed the collection of more fresh blood. This alerted us to the possibility of a bleed producing a neurological deficit. The patient was started on methylprednisolone as per the NASCIS 3 protocol [5]. His coagulation profile was checked and was found to be normal.

**Figure 1:**
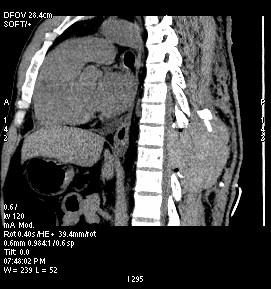
Sagittal view of a computed tomography scan of the dorsal spine showing the hematoma in the extradural space (arrow) compressing the cord and displacing the spinous process.

**Figure 2:**
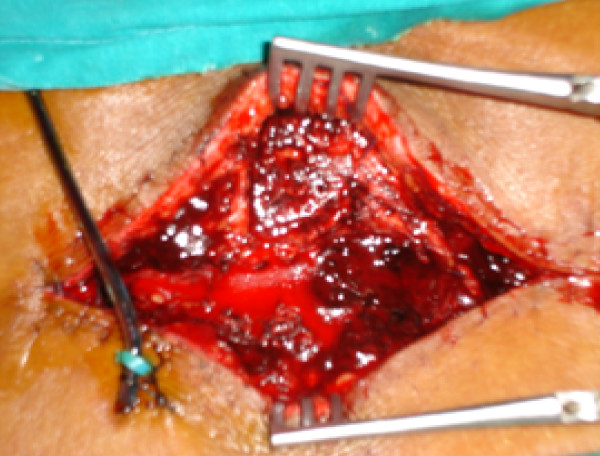
Fresh paraspinal muscular arterial bleed (arrow).

Emergency spinal imaging was advised. Although MRI is the investigation of choice, it could not be carried out due to technical faults in the machinery. Hence, an emergency computed tomography (CT) scan with reconstruction was performed which showed a large extradural hematoma (Figure 1) at the level of the surgical laminectomy. The patient underwent emergency re-exploration 3 hours following the onset of neurological deficit. There was a large hematoma (4.5 cm × 7 cm × 5 cm and approximately 150 ml) on the right side, extending into the epidural space and compressing the cord (Figure 2). The blood clot had pushed away the sutured spinous process superiorly and to the left. The hematoma was evacuated. It was bright red in color suggesting a fresh arterial source. While removing the clot, active arterial spurting from vessels in the right paraspinal muscles was observed at two sites and these were promptly coagulated. No active bleeding from the epidural space was seen. The dura was reopened, but there was no blood collection inside. Neural tissues were found to be normal. Dural closure was performed. Cord pulsation was felt. Spinous processes were removed and paraspinal muscle closure was performed over an epidural drain. A second drain was used in the supramuscular plane. The skin was approximated using subcuticular sutures.

Immediately postoperatively, the patient regained motor power and sensations. He had grade 4 power of both lower limbs and mild paresthesia. He showed good motor and sensory recovery during the following week. Methylprednisolone was continued for 24 hours. He was mobilized cautiously with particular instruction not to strain. He had an uneventful recovery.

## Discussion

The incidence of PSEH is reported to be between 0.1% and 0.2%. Khebaish and Awad [3] have reported only 32 cases in a large series of 14,932 spinal surgeries. Most SEH occurring following spinal surgery are diagnosed within 24 hours. Uribe et al. [4] have reported a series of delayed epidural hematomas in a subset of patients who awoke from surgery neurologically unchanged and then deteriorated more than 3 days following their index procedure. They reported only seven patients with this delayed complication out of 4018 patients studied over a 4-year period. The initial presenting symptom, as in our patient, was sharp pain with radiation to the extremities. Urgent decompression, preferably within 6 hours, aids in neurological recovery [4]. MRI is the investigation of choice but there are instances where MRI cannot be safely performed, as in patients with instrumentation following spinal surgery [1]. CT reconstruction with a high index of suspicion can help in these cases.

Bleeding from Batson's plexus of veins is postulated as a cause of SEH [6]. Tewari and Pandey [7] have suggested that rupture of valveless veins in the internal vertebral plexus, even by the slightest change of posture during sleep, turning or coughing, or due to Valsalva's maneuver, can cause epidural bleeding. However, in our case, the source of bleeding was arterial from paraspinal muscular branches. This is an extraspinal source. The massive clot was causing compression of the dura through the laminectomy defect. We postulate that straining by the patient in the form of paraspinal muscle stretching could have opened up the paraspinal muscular vessels resulting in secondary hemorrhage. This could be due to clot dislodgement from stretching. The rapid progression of the neurological deficit favors arterial bleeding, as was observed intra-operatively. Since the drain had become clearer on the second and third days, the extradural hematoma observed was the result of an acute bleed which could have originated following straining by the patient. Neo et al. [8] have reported a similar case where the patient developed tetraplegia following straining to defecate on the ninth POD following cervical laminoplasty. He had developed a compressive epidural hematoma from arterial bleeding from a split muscle wall.

Patients have to be cautioned against straining themselves following spinal surgeries in the early postoperative period. Careful observation of the drains postoperatively will support suspicions of untoward events. In our case, although a drain was in place, it did not prevent the blood from collecting because no suction was applied. Gravity drains are inferior to suction drains in preventing the collection of blood. The detection of fresh blood in the drain after a period of clear fluid drainage led to the suspicion of an extradural hematoma.

## Conclusion

Delayed SEH is an uncommon cause of neurological deterioration following spinal surgery. Most cases of PSEH occur as a result of venous bleeding. However, an arterial source of bleeding from paraspinal muscular branches is rarely reported. Undue straining by the patient can result in this potentially preventable complication. Clinical evaluation is the most important tool in suspecting such a complication. Fresh blood in the drain during neurological deterioration is an important sign in the clinical detection of PSEH due to acute arterial bleeding. Prompt exploration and decompression gives the best results in these cases.

## Abbreviations

CT: computed tomography; CSF: cerebrospinal fluid; MRI: magnetic resonance imaging; POD: postoperative day; PSEH: postoperative spinal epidural (extradural) hematoma; SHE: spinal epidural (extradural) hematoma.

## Competing interests

The authors declare that they have no competing interests.

## Authors' contributions

Both the authors were involved in the treatment of the above case as well as in the preparation of the manuscript. Both have read and approved the final manuscript.

## Consent

Written informed consent was obtained from the patient for publication of this case report and any accompanying images. A copy of the written consent is available for review by the Editor-in-Chief of this journal.
